# Microbial characterisation and Cold-Adapted Predicted Protein (CAPP) database construction from the active layer of Greenland's permafrost

**DOI:** 10.1093/femsec/fiab127

**Published:** 2021-09-01

**Authors:** Gilda Varliero, Muhammad Rafiq, Swati Singh, Annabel Summerfield, Fotis Sgouridis, Don A Cowan, Gary Barker

**Affiliations:** School of Life Sciences, University of Bristol, 24 Tyndall Ave, Bristol BS8 1TQ, United Kingdom; Centre for Microbial Ecology and Genomics, Department of Biochemistry, Genetics and Microbiology, University of Pretoria, Natural Sciences 2 Building, Private Bag X20, Hatfield 0028, South Africa; Department of Microbiology, Faculty of Life Sciences and Informatics, Balochistan University of Information Technology, Engineering and Management Sciences, Airport Road, Baleli, Quetta, Balochistan, Pakistan; School of Geographical Sciences, University of Bristol, Wills Memorial Building, Bristol BS8 1RL, United Kingdom; School of Life Sciences, University of Bristol, 24 Tyndall Ave, Bristol BS8 1TQ, United Kingdom; School of Chemistry, University of Bristol, Cantock's Cl, Bristol BS8 1TS, United Kingdom; School of Life Sciences, University of Bristol, 24 Tyndall Ave, Bristol BS8 1TQ, United Kingdom; School of Geographical Sciences, University of Bristol, Wills Memorial Building, Bristol BS8 1RL, United Kingdom; Centre for Microbial Ecology and Genomics, Department of Biochemistry, Genetics and Microbiology, University of Pretoria, Natural Sciences 2 Building, Private Bag X20, Hatfield 0028, South Africa; School of Life Sciences, University of Bristol, 24 Tyndall Ave, Bristol BS8 1TQ, United Kingdom

**Keywords:** cold-adapted proteins, soil metagenomics, soil metatranscriptomics, permafrost, Greenland Ice Sheet (GrIS), bioengineering

## Abstract

Permafrost represents a reservoir for the biodiscovery of cold-adapted proteins which are advantageous in industrial and medical settings. Comparisons between different thermo-adapted proteins can give important information for cold-adaptation bioengineering. We collected permafrost active layer samples from 34 points along a proglacial transect in southwest Greenland. We obtained a deep read coverage assembly (>164x) from nanopore and Illumina sequences for the purposes of i) analysing metagenomic and metatranscriptomic trends of the microbial community of this area, and ii) creating the Cold-Adapted Predicted Protein (CAPP) database. The community showed a similar taxonomic composition in all samples along the transect, with a solid permafrost-shaped community, rather than microbial trends typical of proglacial systems. We retrieved 69 high- and medium-quality metagenome-assembled clusters, 213 complete biosynthetic gene clusters and more than three million predicted proteins. The latter constitute the CAPP database that can provide cold-adapted protein sequence information for protein- and taxon-focused amino acid sequence modifications for the future bioengineering of cold-adapted enzymes. As an example, we focused on the enzyme polyphenol oxidase, and demonstrated how sequence variation information could inform its protein engineering.

## Teaser

Permafrost active layer shows similar microbial communities along a proglacial transect in southwest Greenland, and was used to create the Cold-Adapted Predicted Protein (CAPP) database.

## INTRODUCTION

Permafrost is estimated to occur in the 24% of the Northern hemisphere land surface and in the entire Antarctic region (Dobinski 2011). It is one of the most biologically diverse cold-adapted environments where complex and active microbial communities live at constant subzero temperatures (Tuorto *et al*. 2014), and overcome other challenges, such as high salinity, low water and low nutrient availability (Hultman *et al*. 2015). This environment represents a cold and (mostly) dark environment where water is frozen and therefore not readily bioavailable. Permafrost communities generally show high variability in their diversity and structure across different systems where, for example, aerobic and anaerobic heterotrophs, methanogens, sulfate reducers, nitrifying and nitrogen-fixing bacteria are commonly found (Steven *et al*. 2006; Jansson and Taş 2014). Permafrost is enriched in organic carbon content but the latter is not always bioavailable as it is trapped in ice crystals (Jansson and Taş 2014; Schuur *et al*. 2015; Ward *et al*. 2017).

Whereas most of the studies focusing on permafrost communities have investigated microbial community trends in permafrost soil depth gradients and with seasonal changes (Schostag *et al*. 2015; Mackelprang *et al*. 2017; Xue *et al*. 2020), the exploration of permafrost microbial diversity and structure across horizontal patterns have been neglected. However, horizontal microbial trends in other types of soil are well-documented (Huggett 1998), where these trends have been widely shown in proglacial systems (Fernández-Martínez *et al*. 2017; Nash *et al*. 2018; Yoshitake *et al*. 2018). A deeper understanding of permafrost microbial communities is essential because of the important role that these organisms could play in permafrost thawing induced by global warming where, particularly the respiration of newly released and biologically available organic carbon, could further increase the emission of greenhouse gases (Ganzert *et al*. 2007; Mackelprang *et al*. 2011; Makhalanyane *et al*. 2015). These microbial communities are also important from a biodiscovery perspective (Jansson and Taş 2014).

Studies of bacterial isolates and shotgun metagenomic DNA sequences obtained from permafrost have been widely used in the biodiscovery of cold and stress adaptation and, in particular, cold-adapted enzymes (Vishnivetskaya and Kathariou 2005; Bakermans *et al*. 2009; Ayala-Del-Río *et al*. 2010; Mykytczuk *et al*. 2013; Collins and Margesin 2019). The use of cold-adapted proteins is potentially advantageous in several industrial and medical settings (e.g. food and pharmaceutical industries) because their enzymatic reactions do not require excessive heating, being in this way more energetically sustainable, giving high reaction yields and fewer unwanted secondary chemical reactions at low temperatures (Siddiqui 2015; Kaur and Gill 2019; Mangiagalli *et al*. 2020). The use of cold-adapted enzymes is also useful for bioremediation processes where the direct use of purified enzymes in cold settings can degrade toxic compounds such as phenolic substances, hydrocarbons, plastics and pesticides both *in situ* and *ex situ* (Karigar and Rao 2011; Sharma, Dangi and Shukla 2018; Kumar, Kumar and Giri 2019).

Protein engineering can be used to enhance the stability and activity of native proteins. This can be achieved by induced random protein mutations or targeted amino acid substitutions (Kano, Taguchi and Momose 1997; Kryukova *et al*. 2019). Whereas the first approach requires a wide and expensive screening of all the randomly produced proteins, the second is cheaper as relies on the exploration of homologous protein alignments to retrieve functional amino acid substitutions. Targeted amino acid substitutions are effectively applied when sequences homologous to the studied proteins are present in online or custom protein databases. High-throughput (e.g. Illumina) and long read (e.g. nanopore) sequencing technologies can yield complete Metagenome-Assembled Genomes (MAGs) where genes, gene clusters and predicted proteins can be assigned with high reliability (Somerville *et al*. 2019; Stewart *et al*. 2019). This facilitates the mining of metagenomic and metatranscriptomic datasets for new gene products and protein sequences, enriching public databases with protein sequences obtained from a broad range of organisms and environments. In this study, we focused on the enzyme polyphenol oxidase. This enzyme catalyses the conversion of phenols to non-toxic quinines, and therefore could be used for the treatment of phenolic polluted environments (Babich and Davis 1981; Panadare and Rathod 2018).

We collected a total of 102 different permafrost active layer soil samples from 34 different locations in the Greenland Ice Sheet (GrIS) proglacial system, in an area extending from the ice edge to Kangerlussuaq, where permafrost has been previously reported (Van Tatenhove and Olesen 1994; Jørgensen and Andreasen 2007; Clarhäll 2011; Johansson *et al*. 2015). These samples were collected across a wide range of environments (i.e. riverbanks, thermokarst bogs, grasslands and heath-dominated environments). DNA extracted from all the samples was sequenced using both nanopore and Illumina technologies to facilitate a reliable and complete assembly (Bertrand *et al*. 2019; De Maio *et al*. 2019), leading to the recovery of MAGs, the prediction of full open reading frames (ORFs), the annotation of full-length genes and predicted proteins, and the exploration of microbial community compositions. Whole shotgun sequencing data was combined with metatranscriptomic data and a geochemical characterisation of all samples to identify the active portion of the permafrost microbial community, and the soil properties at which the gene transcripts were more likely transcribed at subzero temperatures.

This study had two main aims. The first aim was the characterisation of the active layer community in the GrIS proglacial system, focusing on microbial DNA and cDNA profiles. In particular, we explored whether the microbial communities were shaped by their distance from the ice edge, as observed for surface soil microbial communities in proglacial systems (e.g. Yoshitake *et al*. 2018); or by soil geochemistry; or whether the communities were consistent across the different sites, being shaped mainly by the challenging conditions set by permafrost environment. In the second part of this study, we used the deep-sequence (>164x) assembly to recover highly complete genomes and predict the sequences of more than three million cold-adapted proteins, from which we constructed a Cold-Adapted Predicted Protein (CAPP) database. Focusing on the enzyme polyphenol oxidase, we also showed how the CAPP database can be used to inform protein engineering approaches.

## MATERIALS AND METHODS

### Sampling area and sample collection

**Figure 1.**
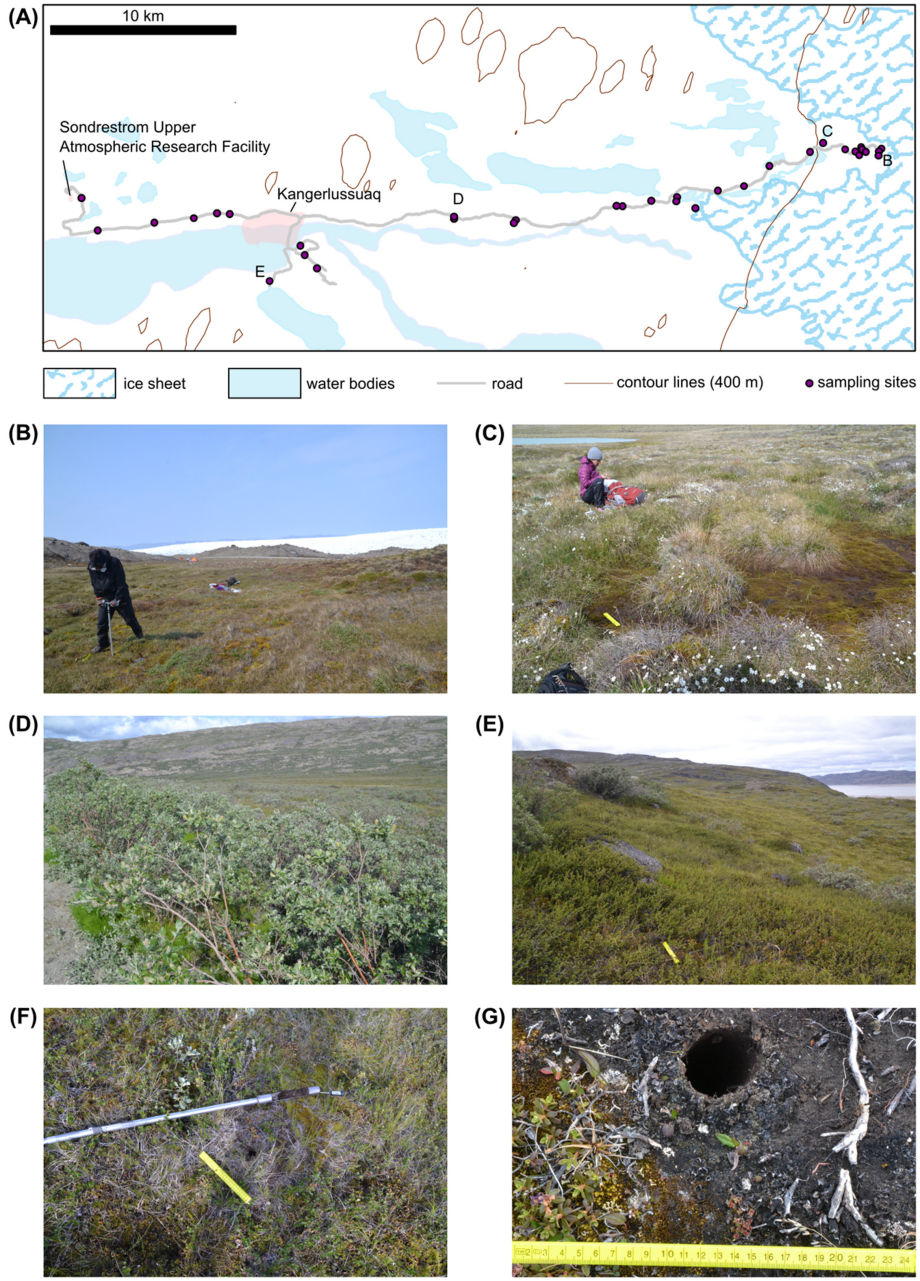
Overview of the ice sheet proglacial system sampling sites **(A)**. The map also indicates where images B, C, D, and E were taken. Sites defined as grassland were dominated by grass (e.g. *Calamagrostis langsdorfii*) and moss **(B)**. Wetland was close to lakes, rivers and bogs where there was a dominance of grass, dwarf-shrubs, *Eriophorum scheuchzeri*, *Poa alpina* and *Poa pratensis***(C)**. *Salix* heath was dominated by high-shrubs of the genus *Salix***(D)**. Vegetation defined as dwarf-shrub heath was dominated by *Betula nana*, *Rhododendron lapponicum*, *Vaccinium uliginosum* and *Ledum palustre***(E)**. All the soil samples were taken with 38 mm diameter soil corer **(F-G)**.

In July 2018, 102 soil core samples were collected from 34 locations along a 43 km transect in the Kangerlussuaq area, Greenland. The area was selected for logistical practicality (proximity to the Kangerlussuaq International Science Station and airport, and good road access) and because previous field work showed that permafrost was readily accessible using manual corers. Samples were collected following the road which connects the ice edge (point 660), Kangerlussuaq and the Sondrestrom Upper Atmospheric Research Facility (Fig. 1A), forming a transect from the glacial ice edge in the direction to the Greenland coastline. Because the GrIS proglacial field is a complex system where the ice moves and retreats in different directions, sample distance from the ice edge was calculated as the distance to the closest ice edge point (Appendix A: Table A1).

We collected frozen soil samples from the upper permafrost layer (i.e. active layer) of a variety of habitats and overlying vegetation-types: riverbanks, thermokarst bogs, grasslands and from heath-dominated environments (Fig. 1B-E). The site vegetation was characterised following Clarhäll (2011) . We categorized the sites into four different vegetation types: (i) “grassland”, which is dominated by grass (e.g. *Calamagrostis langsdorfii*) and moss (Fig. 1B); (ii) “wetland”, in soils on the margins of lakes, rivers and bogs which are dominated by grass and dwarf-shrubs, including *Eriophorum scheuchzeri*, *Poa alpina* and *Poa pratensis* (Fig. 1C); (iii) “*Salix* heath” which is dominated by high shrubs belonging to *Salix* species (Fig. 1D) and (iv) “dwarf-shrub heath”, dominated by *Betula nana*, *Rhododendron lapponicum*, *Vaccinium uliginosum* and *Ledum palustre* (Fig. 1E).

Sampling took place in July 2018, with an average air temperature of 6.1°C over the month (average temperature of the other months ranged from -24.3°C to 4.8°C; data from https://power.larc.nasa.gov/). During the warmest months, the snow melts and the thawing front is deeper in the soil (Hayashi, Van Der Kamp and Schmidt 2003; Elberling *et al*. 2008). Therefore the sampled soil, being at subzero temperature in July, was likely at subzero temperature all year round. During the sampling activity it rained every day with precipitation values ranging between 0.4 and 10.3 mm day^–1^ (data from https://power.larc.nasa.gov/). For this reason, the soil temperature of several sampled sites was checked on different days; the presence of frozen soil did not vary even after heavy rains occurred and the soil temperature only varied of ±0.2°C. Samples were collected with a 38 mm diameter soil corer (https://www.geopacks.com/products/soil-sampling-corer) (Fig. 1F-G) which was modified with an extended shaft so that it could reach two-meters depth. At each site, three core samples were collected within a distance of 20 meters. The soil samples were all collected between 30 and 92 cm depth (Appendix A: Table A1). The sampling depth variation was due to different soil textures (Appendix A: Table A2) observed in the different areas, where a wide range of soil depths between the surface and the bedrocks was also present. Soil temperatures were measured with a portable digital thermometer at the bottom of the soil core immediately after sample collection, and ranged between -0.1°C and -2°C (Appendix A: Table A1). Soil cores were discarded if the measured temperature was above 0°C.

The soil was always collected at the end of the soil core where also the core temperature was measured. For each sample, soil was collected both for geochemical analyses and nucleic acid co-extraction. For geochemical analyses, the soil was collected in a 15 mL Falcon tube. For nucleic acid co-extraction, one gram of soil was sampled; soils coming from the same site were pooled together (for a total of 3 grams per site) in a 15 mL Falcon tube and preserved in 2x LifeGuard Soil Preservation solution (QIAGEN, Hilden, Germany). All samples were kept chilled in a portable cool box and then frozen at -20°C in the Kangerlussuaq International Science Support (KISS) station within two days of collection.

### Geochemical analyses

Soil samples were thawed at 4°C, manually homogenised, and all visible roots were separated by sieving (2 mm). Subsequent analyses were performed on the sieved fraction (< 2 mm). Root biomass and soil moisture content were determined gravimetrically by drying at 105°C for 24 hours in a convection oven. Organic matter content was determined by loss on ignition in a muffle furnace after heating 1 g of dried soil at 550°C for 4 hours. Following treatment of soils with loss on ignition (to remove organic matter), the absolute particle size distribution of the mineral soil fraction was determined with optical laser diffraction using a MS3000 Mastersizer (Malvern Instruments Ltd., UK).

Field-moist soils (1 g) were extracted at a ratio of 5:1 with 5 mL 2 M KCl for the determination of exchangeable ammonium (NH_4_^+^). 5 g samples were extracted with 25 mL of deionised water for the determination of dissolved nitrate (NO_3_^–^), phosphate (PO_4_^3–^), silicon (Si), total iron (Fe), major anions and cations and Dissolved Organic Carbon (DOC). The soil slurries were continuously shaken on a reciprocating shaker at 200 rpm for 1 hour before being centrifuged at 5,000rpm for 10 minutes followed by filtration of the supernatant extract with 0.22 µm pore size PES syringe filters (25 mm diameter). Ammonium was analysed spectrophotometrically on a Gallery-Plus Automated Photometric Discrete Analyser (Thermo Fisher Scientific, UK) using a salicylate-hypochlorite alkaline reaction method with absorbance measured at 660 nm; nitrate using a hydrazine-sulfanilamide reaction method with absorbance measured at 540 nm; phosphate using the molybdenum blue method with absorbance measured at 880 nm; silicon using an ammonium molybdate – ascorbic acid reaction method with absorbance measured at 700 nm; and total iron using a hydroxylamine-ferrozine reaction method with absorbance measured at 562 nm. The limits of detection were 0.01 mg N, P, Si or Fe L^–1^, the samples were blank corrected, while the precision as a Relative Standard Deviation (RSD) was < 2%. Soil pH was also measured in the deionised water extracts using the Gallery Plus built-in probe (calibrated 4-7 pH).

DOC concentrations were quantified using a Shimadzu TOC-L Organic Carbon Analyzer, with a high salinity module. Non-Purgeable Organic Carbon (NPOC) was measured after acidification of samples with 9 M H_2_SO_4_ and catalytic combustion (680°C) of dissolved organic carbon to carbon dioxide, which was then measured by infrared absorption. The limit of detection was 0.01 mg C L^–1^, the samples were blank corrected, while the precision was < 5% RSD. For the simultaneous determination of Total Nitrogen (TN) and Total Phosphorus (TP), an aliquot (5 mL) of the deionised water soil extracts was digested using the potassium persulfate oxidation method of Johnes and Heathwaite (1992) modified for the CEM MarsXpress microwave digestion unit. TN and TP were measured spectrophotometrically as nitrate and phosphate, respectively as described above. Dissolved Organic Nitrogen (DON) and Dissolved Organic Phosphorus (DOP) were calculated by difference (DON = TN - NO_3_^–^ - NH_4_^+^ and DOP = TP - PO_4_^3–^).

Sulphate and major cations (Na^+^, K^+^, Ca^2+^ and Mg^2+^) were measured simultaneously in the deionised water soil extracts using an ICS5000 ion chromatograph (Thermo Fisher Scientific, UK). Sulphate was separated isocratically from other anions on an AS11-HC 2-mm column at 0.25 mL min^–1^ flow rate using 24 mM KOH eluent. Cations were separated isocratically on a CS12 2 mm column at 0.25 mL min^–1^ flow rate using 20 mM MSA eluent. The limit of detection was 0.001 mg L^–1^ for all measured anions and cations, the samples were blank corrected, while the precision was < 2% RSD.

### DNA preparation and sequencing

DNA and RNA were coextracted using the RNeasy PowerSoil Total RNA Kit and RNeasy PowerSoil DNA Elution Kit (QIAGEN) according to manufacturer's instructions. The soil, preserved in LifeGuard Soil Preservation solution (QIAGEN), was centrifuged, the liquid was discarded, and the soil was divided for two extractions (1.5 g per extraction). Roots were excluded from the soil where possible. Extracted DNA and RNA concentrations were checked with Qubit^®^ Fluorometer, Qubit^®^ dsDNA HS assay kit and Qubit^®^ RNA HS assay kit (Invitrogen, Carlsbad, CA, USA) (Appendix B: Table B1 and B2).

Extracted DNA was treated with DNase-free RNase A (Sigma-Aldrich, Darmstadt, Germany) for 30 minutes at 37°C, purified using phenol:chloroform:isoamyl alcohol (25:24:1) and precipitated with 100% ethanol. RNA was purified with the TURBO DNA-free kit (Thermo Fisher Scientific, Carlsbad, CA, USA) and concentrated with RNeasy MinElute Cleanup (QIAGEN). SuperScript IV Reverse Transcriptase (Thermo Fisher Scientific) was used to synthesize the first cDNA strand, and the SuperScript Double-Stranded cDNA Synthesis Kit protocol (Thermo Fisher Scientific) was used for the synthesis and the successive clean-up of double-stranded cDNA.

Both DNA and cDNA were sequenced with the MinION device (Oxford Nanopore Technologies, Oxford, UK). Per flow cell (FLO-MIN106), between 4 and 6 samples were barcoded together with the Native Barcoding Kit EXP-NBD104 (Oxford Nanopore Technologies); sequencing libraries were prepared with the Ligation Sequencing Kit SQK-LSK109 (Oxford Nanopore Technologies). Two DNA samples were also sequenced with the Illumina technology: sample 34, chosen because of the high sequencing yield obtained with nanopore sequencing (14,228,430,364 bases), and a sample composed by all the other samples pooled together. Illumina libraries were prepared with the Illumina Nextera chemistry v 3 (Illumina, San Diego, CA, USA) and 150 bp paired-end sequences were obtained using the NovaSeq platform (Macrogen, Seoul, South Korea). Basecalling was performed with the Real Time Analysis (RTA) software v 3.3.3.

The raw nanopore and Illumina fastq files were deposited in the European Nucleotide Archive and can found at http://www.ebi.ac.uk/ena/data/view/PRJEB42267.

### Bioinformatics analyses

Nanopore sequences were basecalled and adapter trimmed with Guppy v 3.2.2 (Oxford Nanopore Technologies). All nanopore sequences longer than 1,000 bases were assembled with metaFlye (Flye v 2.7) (Kolmogorov *et al*. 2020). Nanopore reads were then mapped to the assembly with minimap2 v 2.17 (Li 2018) and used to polish the assembly with one cycle of Racon v 1.4.15 (Vaser *et al*. 2017) using the options ‘-m 8 -x -6 -g -8 -w 500’ (same options used to train the neural network polishing software medaka), and one cycle of medaka v 0.10 (https://github.com/nanoporetech/medaka). The assembly was then polished with the Illumina reads, with four cycles of Pilon v 1.23 (Walker *et al*. 2014). Before each Pilon step, reads were mapped back to the assembly with bwa v 0.7.17 with the mem algorithm (Li and Durbin 2010) where the percentage of mapped reads increased from 67% to 69%, with an increase of more than 34 million reads from the beginning of the polishing steps.

Contig binning was performed with five different algorithms: MetaBAT v 2.12.1 (Kang *et al*. 2019), MaxBin v 2.2.7 (Wu *et al*. 2014), CONCOCT v 1.1.0 (Alneberg *et al*. 2014), BinSanity v 0.3.8 (Graham, Heidelberg and Tully 2017) and DAS Tool v 1.1.1 (Sieber *et al*. 2018), which combines the information obtained from all the other binning software and therefore was used to ultimately define the bins. All the bins (MAGs) were checked with CheckM v 1.1.2 (Parks *et al*. 2015). High-quality MAGs were defined as bins with a completeness greater than 90% and less than 5% contamination. High-quality MAGs, together with the assembly, were uploaded the European Nucleotide Archive (ENA) (http://www.ebi.ac.uk/ena/data/view/PRJEB42267). Medium-quality MAGs were defined with a threshold of 50% and 10% for completeness and contamination, respectively. These thresholds were defined in different published works (Bowers *et al*. 2017; Almeida *et al*. 2019; Stewart *et al*. 2019).

Coding regions were assigned to the polished assembly with Prokka v 1.14.6 (Seemann 2014). This software uses Prodigal v 2.6.3 for Open Reading Frame (ORF) prediction (Hyatt *et al*. 2012), barrnap v 0.9 for ribosomal RNA prediction (https://github.com/tseemann/barrnap) and ARAGORN v 1.2.38 to predict tRNA and tmRNA coding genes (Laslett and Canback 2004). Secondary metabolite gene clusters were predicted using antiSMASH v 5.1.2 (Blin *et al*. 2019) on high- and medium-quality MAGs. Taxonomy was assigned to the assembled contigs using Diamond v 0.9.22 (Buchfink, Xie and Huson 2015) and the NCBI non-redundant (nr) database v 5 (ftp://ftp.ncbi.nih.gov/blast/db/FASTA/nr.gz) (Sayers *et al*. 2020). The Diamond output was then used as input to the LongMeta pipeline to obtain taxonomy annotation associated with each assembled contig https://github.com/gvMicroarctic/LongMeta. Nanopore reads (i.e. DNA and cDNA reads) were then mapped back to the assembly with minimap2 v 2.17 (Li 2018) in order to obtain taxonomy profiles associated to each sample. Taxonomy profiles were analysed for both the metagenomic and metatranscriptomic data sets. The first were analysed with the LongMeta pipeline where all the commands were run with default parameters except for longMeta-coverage where –perc-limit was set to 0.001. DNA-based taxonomy trends were estimated as coverages, while cDNA-based taxonomy trends were estimated as read counts associated to each different taxonomic assignment.

### Cold-Adapted Predicted Protein (CAPP) database

The coding regions and proteins predicted from the high-quality assembly were used to construct the Cold-Adapted Predicted Protein (CAPP) database. Genes coding for rRNA, tRNA and tmRNA were excluded from the database. A list of the predicted proteins and coding regions, and all information associated to each entry of the CAPP database can be found at the GitHub page https://github.com/gvMicroarctic/CAPP_database. Nanopore cDNA sequences were mapped to CAPP genes with minimap2 v 2.17 (Li 2018). Proteins from the National Center for Biotechnology Information (NCBI) non-redundant protein database (nr; https://www.ncbi.nlm.nih.gov/) were mapped to the CAPP proteins with Diamond v 0.9.22 (Sayers *et al*. 2020).

CAPP amino acid frequencies were compared to protein sequences associated to 10 known thermophilic and 10 known psychrophilic genomes (Appendix C: Table C1). The data were downloaded from the NCBI database (https://www.ncbi.nlm.nih.gov/genome/). The amino acid frequencies were then calculated on the CAPP database, CAPP protein assigned to medium- and high-quality MAGs, and proteins from the NCBI reference genomes.

For the tree construction, only the CAPP protein sequences assigned to PolyPhenol Oxidase (PPO; EC 1.10.3.-) and that mapped back to at least one nanopore cDNA read were retrieved. These proteins, together with the nr proteins that aligned to them, were then aligned with mafft v 7.271 (Katoh and Standley 2013) and then used to construct a phylogenetic tree with iqtree v 1.6.12 (Nguyen *et al*. 2015). The newick tree was then drawn as cladogram with the software FigTree v 1.4.4 (http://tree.bio.ed.ac.uk./software/figtree/). This tree was built to show the sequence relation and clustering without any purpose to show evolutionary relationships between the different protein clusters.

To identify potential PPO conserved motifs in PPO cold-adapted variants, Position-Specific Iterated BLAST (PSI-BLAST) (Altschul *et al*. 1997) was used to identify PPO sequences from differently thermo-adapted PPO enzymes. The PSI-BLAST algorithm detects sequences that are distantly related to our query sequence allowing us to identify sequence from mesophilic (WP_028524802.1, WP_183811557.1, HBQ27707.1) and thermophilic bacteria (WP_060635499.1, MBK9154733.1, WP_147647240.1, WP_015255161.1, WP_120166071.1). Once these meso- and thermophilic sequences were retrieved, multiple sequence alignment was performed along with the CAPP sequences retrieved from the Acidobacteria cluster highlighted in PPO phylogenetic tree.

### Statistical analyses

All the statistical analyses and the result plots were performed in the R environment v 6.6.1 (R Core Team 2019) including the R packages vegan v 2.5-6 (Oksanen 2017), gplots v 3.0.3 (Warnes 2012), ggplot2 v 3.3.0 (Wickham 2016), ggfortify v 0.4.10 (Tang, Horikoshi and Li 2016), tidyr v 1.0.3 (Wickham and Henry 2019), plyr v 1.8.6 (Wickham 2011) and gridExtra v 2.3 (Auguie and Antonov 2017).

Mantel tests were performed to analyse the relation between site distance from the ice edge, geochemical variables and taxonomic trends. Mantel test statistics (*r*) were calculated with 9,999 permutations and considered significant only for *P*-values < 0.05. *P*-values were calculated using two-tailed tests. Mantel tests were calculated using Spearman's rank correlation coefficients on Euclidean matrices when considering the distance from the ice edge and the geochemical dataset, and Bray-Curtis matrices for the taxonomic datasets. Spearman's rank-order correlation coefficient (*r*_s_) was calculated to i) detect correlations between the distance from the ice edge and the other geochemical variables and ii) detect correlations between the DNA and cDNA datasets and the geochemical variables. The *r*_s_ was considered significant only if *P*-value < 0.05. All taxonomic profiles are reported as relative abundances calculated on coverage values for DNA dataset and as read counts for the cDNA dataset.

## RESULTS

### Site characterisation

A positive and significant correlation between both soil geochemistry (*r* = 0.21, *P*-value < 0.05, *n* = 102) and grain size (*r* = 0.32, *P*-value < 0.05, *n* = 102), and distance from the ice edge, was identified. Spearman's correlation analyses showed a significant positive correlation (*P*-value < 0.05) between distance from the ice edge and the vegetation complexity, soil pH, root weight percentage, PO_4_^3–^, Na^+^ and total Fe concentration, and a significant negative correlation with altitude, soil temperature, organic matter percentage, TN, NO_3_^–^, SO_4_^2–^, and Ca^2+^ concentration. The other geochemical factors tested did not show any significant correlation associated with the distance from the ice edge (Table 1 and Appendix D). Grain size data and geochemical values are reported in Appendix A (Tables A2 and A3).

**Table 1.** Spearman's rank correlation coefficients (r_s_) calculated between the site distance from the ice edge and site variables (n = 102). *in the definition of vegetation complexity the grassland and wetland environments were assumed to be the least complex, followed by dwarf-shrub heath and Salix heath. **grain size was defined as the peak size value in the grain size distribution.

Site and geochemical factors	*r*_s_	*P*-value
altitude	−0.8206	0.0000
vegetation*	0.3447	0.0004
soil temperature	−0.1987	0.0040
pH	0.4626	0.0000
soil depth	0.0822	0.4113
soil moisture	−0.1599	0.1084
root	0.1997	0.0442
organic content	−0.2674	0.0066
grain size**	−0.1158	0.2464
TN	−0.2345	0.0177
DON	−0.1720	0.0840
TP	−0.0245	0.8070
DOP	−0.0510	0.6107
DOC	0.0679	0.4975
NO_3_^-^	−0.3751	0.0001
NH_4_^+^	−0.1232	0.2173
PO_4_^3-^	0.3210	0.0010
SO_4_^2-^	−0.2406	0.0148
Na^+^	0.2693	0.0062
K^+^	0.1479	0.1380
Mg^2+^	−0.1554	0.1189
Ca^2+^	−0.3875	0.0001
total Fe	0.2608	0.0081
Si	−0.0390	0.6973

### Assembly result and MAG reconstruction

Between 139,787,928 (sample 32) and 14,228,430,364 bp (sample 34) of sequence data were obtained from DNA samples sequenced with nanopore sequencing technology (Appendix B: Table B3). A total of 800 gigabases of data was obtained from the Illumina sequencing. The final assembly, constructed with nanopore sequences and polished with Illumina reads, consisted of 2,834,571,546 bp. The assembly N50 was 40,615 bases, the minimum contig length was 1,000 bases, and the longest contig was 3,693,048 bp long. The latter corresponded to a complete and circularized bacterial genome (MAG-01). Illumina and nanopore base coverage in the assembly was 164x and 18x, respectively. The different binning algorithms resulted in different numbers of recovered bins: Metabat2 yielded 1,005 bins, MaxBin2 517, Concoct 236, BinSanity 618 and DAS Tool 267. DAS Tool, which combines all the information obtained from the other tools to create consistent bins, yielded nine high-quality MAGs (completeness ≥ 90 and contamination ≥ 5) and 60 medium-quality MAGs (completeness ≥ 50 and contamination ≥ 10).

All the high-quality MAGs had consistent taxonomy across all the contigs, and belonged to the kingdom Bacteria. The high-quality MAGs belonged to the phyla Chloroflexi (four MAGs), Acidobacteria (two MAGs), Proteobacteria (two MAGs) and Bacteroidetes (one MAG). MAG-01 was a complete MAG, circularized and ungapped genome, belonging to the bacterial family Geobacteraceae (Table 2). Medium-quality MAGs were also assigned to the phyla Chloroflexi (six MAGs), Acidobacteria (eight MAGs), Proteobacteria (11 MAGs) and Bacteroidetes (four MAGs), Actinobacteria (14 MAGs), Verrucomicrobia (two MAGs) and other phyla such as Gemmatimonadetes and Nitrospirae. MAG-20 was the only one assigned to Archaea (phylum Thaumarchaeota) with a completeness of 85% and a contamination of 5% (Appendix C: Table C2). Only ten high- and medium-quality MAGs were assigned at the family or genus level.

**Table 2.** High-quality MAGs. The table reports MAG completeness, contamination and strain heterogeneity as calculated from CheckM. It also reports the number of contigs in each MAG and the taxonomic classification as phylum, class, order and family. The taxonomic classification was reported only when more than the 70% of the contigs were assigned to the same taxon.

MAGs	Completeness	Contamination	Strain heterogeneity	Contig number	Taxonomy
MAG-01	99.6	0.0	0.0	1	Proteobacteria, Deltaproteobacteria, Desulfuromonadales, Geobacteraceae
MAG-02	99.1	0.9	0.0	5	Chloroflexi
MAG-03	98.2	0.9	100.0	5	Chloroflexi
MAG-04	98.2	4.6	16.7	19	Chloroflexi
MAG-05	96.8	3.9	50.0	129	Acidobacteria
MAG-06	96.0	3.2	0.0	30	Proteobacteria
MAG-07	96.0	1.0	100.0	11	Chloroflexi
MAG-08	91.0	1.3	25.0	25	Bacteroidetes, Chitinophagia, Chitinophagales, Chitinophagaceae
MAG-09	90.2	4.9	0.0	138	Acidobacteria

The assembly was annotated with Prodigal which identified 3,109,960 open reading frames (ORFs). Almost 99% of these were CDSs (Coding DNA Sequences), 1% were rRNA genes and less than 0.1% were represented by tRNA and tmRNA genes. About 16% of the ORFs could be mapped back to the cDNA transcripts. Between 45 and 84% and 46 and 83% of the nanopore DNA reads (≥ 1,000bp) and cDNA reads (≥ 100 bp) mapped back to the assembly (Appendix B: Tables B3 and B4); whereas 80-84% of the Illumina reads mapped back to the assembly (Appendix B: Table B5).

### Metagenomic trends

Mantel test statistics (*r*) showed no correlation between the taxonomic composition and the distance from the ice edge. Mantel test statistics (*r*) were, however, significant (*P*-value < 0.05) between the taxonomic datasets at phylum-, class- and genus-level and geochemical dataset (*r* = 0.20, *r* = 0.22, *r* = 0.22 respectively; *n* = 34).

**Figure 2.**
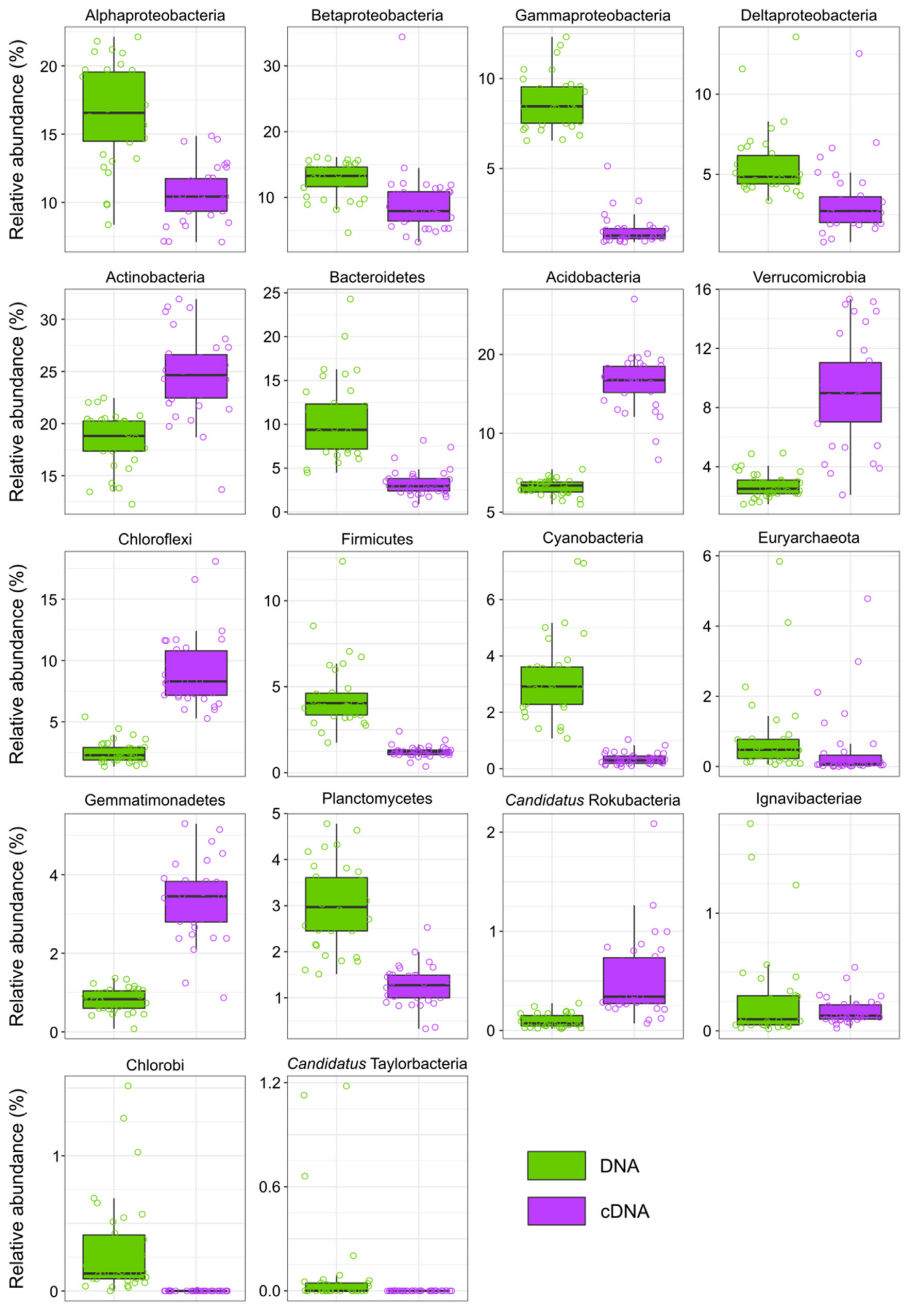
Most abundant phyla and Proteobacteria classes in the DNA and cDNA profiles. Only taxa with a minimum relative abundance of 1% in at least one sample of the DNA or cDNA datasets are reported.

Bacteria represented between 92.7% and 99.7% of the taxa in all samples. Archaea represented between 0.1 and 7.2%, viruses between 0.0% and 1.3% and Eukaryota, all belonging to fungal taxa, were present at < 1.5%in all samples (Appendix E: Table E1). Microbial distribution did not show significant trends along the sampling transect (Appendix E). The most abundant phyla were Proteobacteria (33.2%-51.8%), followed by Actinobacteria (12.3%-22.4%), Bacteroidetes (4.5%-24.3%), Firmicutes (1.7%-12.3%), Cyanobacteria (1.1%-7.3%), Planctomycetes (1.5%-4.8%), Chloroflexi (1.4%-5.4%), Acidobacteria (1.0%-5.4%) and Verrucomicrobia (1.4%-4.9%). At the class level, Alphaproteobacteria were present at 8.4%-22.1%, Betaproteobacteria at 4.6%-15.7%, Deltaproteobacteria at 3.4%-13.6%, and Gammaproteobacteria at 6.6%-12.3% (Fig. 2). Other bacterial classes present at high relative abundances were Actinobacteria (10.6%-20.0%), Chitinophagia (0.9%-9.9%), Clostridia (0.7%-9.2%) and Bacteroidia (0.2%-12.7%). The most abundant archaeal phyla were Euryarchaeota representing up to the 5.8% of the community in the samples, and Thaumarchaeota, representing up to 1.6%.

### Metatranscriptomic trends

The nanopore cDNA sequencing output ranged between 4,302,632 bp (sample 7) and 1,021,029,268 bp (sample 24) (Appendix B: Table B4). In each sample, between 87 and 98% of the nanopore cDNA reads that mapped back to the assembly, fell inside predicted genes. Of these, more than 98% of the mapped cDNA reads spanned across only a single ORF whilst 2% spanned two or more ORFs. In almost all the samples, more than 98% of the mapped reads were represented by genes coding for ribosomal RNA. The exceptions were samples 5, 18 and 25 were the ribosomal reads only represented 94, 91 and 57% of the reads, respectively.

Mantel test statistics (*r*) showed no correlation between cDNA taxonomy and site distance. However, the correlation was significant between the geochemical dataset and the cDNA-based taxonomy with an *r* equal to 0.23, 0.36, 0.40 at phylum-, class- and genus-levels respectively (*P*-value < 0.05, *n* = 34).

In this dataset, the most abundant phyla were the same as in the metagenomic dataset. A consistent microbial community was observed across sites (Appendix E), and no phyla (or Proteobacteria classes) showed a significant correlation with the distance from the ice edge (data not shown). However, the relative abundances varied between DNA and cDNA datasets. Proteobacteria varied between 13.5% and 53.0%, Actinobacteria between 13.7% and 31.9%, Acidobacteria 6.6% and 27.0%, Chloroflexi 5.3% and 18.1%, Verrucomicrobia 2.1% and 15.3%, Bacteroidetes 0.9% and 8.2%, Gemmatimonadetes 0.9% and 5.3%, Planctomycetes 0.3% and 2.5%, Firmicutes 0.4% and 2.4%, *Candidatus* Rokubacteria 0.1% and 2.1%, and Cyanobacteria 0.1% and 1.0%. The most abundant classes were Betaproteobacteria (3.2%-34.4%), Actinobacteria (13.1%-28.7%), Alphaproteobacteria (7.1%-14.6%), Deltaproteobacteria (0.8%-12.5%), Gammaproteobacteria (1.0%-5.1%), and Chitinophagia (0.6%-2.9%) (Fig. 2 and Appendix E).

### Microbial dataset and site characteristic comparison

Mantel test statistics (*r*) showed a significant (*P*-value < 0.05) correlation between the taxonomic DNA and cDNA datasets at phylum-, class- and genus-level (*r* = 0.33, *r* = 0.48, *r* = 0.51; *n* = 34). Comparing the most abundant phyla (i.e. minimum relative abundance of 1% in at least one sample) between the DNA and cDNA datasets, the phyla Actinobacteria, Acidobacteria, Verrucomicrobia, Chloroflexi, Gemmatimonadetes and *Candidatus* Rokubacteria were the only ones present with a higher abundance in most of the sites for the cDNA dataset (Fig. 2).

**Figure 3.**
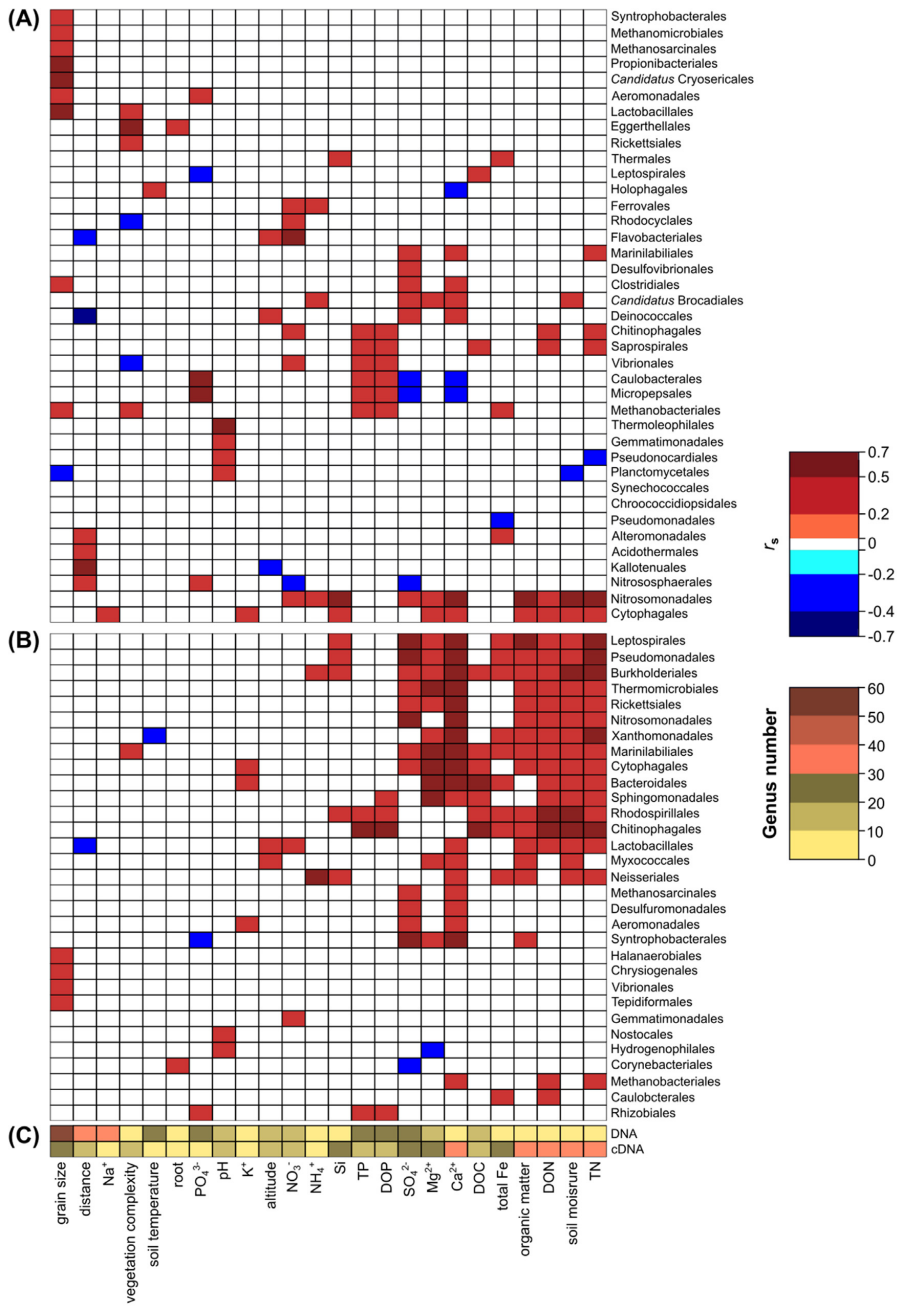
Taxonomic correlation with site characteristics and geochemical variables. Spearman's rank correlation coefficients (*r*_s_) between taxonomic orders that significantly correlated (*P*-value < 0.05; *n* = 34) with at least one of the site variables for DNA **(A)** and cDNA **(B)**. Number of genera that showed a significant correlation (*P*-value < 0.05) in the DNA or cDNA dataset **(C)**.

Compared to the DNA dataset, the cDNA dataset presented more bacterial taxa (order-level) that showed significant (*P*-value < 0.05) Spearman's correlation coefficients (*r*_s_) in relation to different geochemical variables (Fig. 3). In the cDNA dataset, several bacterial orders showed a positive correlation with the ion and nutrient distributions. In particular, the microbial taxonomic orders Leptospirales, Pseudomonadales, Burkholderiales, Thermomicrobiales, Rickettsiales, Marinilabiales and Cytophagales showed a positive correlation with Ca^2+,^ Mg^2+,^ SO_4_^2–^, DON and TN concentrations, soil moisture and percent organic matter. Further, Burkholderiales, Marinilabiales, Cytophagales, Bacteroidales, Sphingomonadales, Rhodospirillales and Chitinophagales also showed positive correlation with DOC. No correlations between cDNA relative abundances and the distance from the ice edge were observed except for Lactobacillales which showed negative correlation (Fig. 3B).

When the Spearman's correlation coefficient (*r*_s_) between genus-level taxonomic diversity and the biochemical/physical parameters was calculated, the DNA and the cDNA datasets showed significant *r*_s_ for 225 and 116 genera (out of 941 and 860 in total), respectively. More genera in the DNA dataset correlated with the distance from the ice edge and other site-based characteristics, including altitude, soil temperature and grain size, and also TP, DOP, NO_3_^–^, PO_4_^3–^, Na^+^ and K^+^, compared to the cDNA dataset. In the cDNA dataset, more genera (280) positively correlated with vegetation, soil moisture, organic content, TN, DON, DOC and ion concentrations, such as NH_4_^+^, SO_4_^2–^, Mg^2+,^ Ca^2+,^ total Fe and Si (Fig. 3C).

### CAPP database exploration

The CAPP database consisted of 3,076,838 coding regions/proteins. The metadata associated with each entry comprise of the entry name, the predicted gene and protein, the related Enzyme Commission (EC) number and how the coding region was assigned (i.e. by similarity to protein motif or sequence). It reports the taxonomy assignment and the MAG name in case the predicted protein originated from one of the high-quality or medium-quality MAGs, and whether the protein coding sequence matched to one or more cDNA reads (516,863 predicted proteins were matched by at least 1 cDNA read). Further, metadata information also reports if there were CAPP matches to the National Center for Biotechnology Information (NCBI) non-redundant protein database. The CAPP database also associates geochemical information to each CAPP entry that was found expressed in the environmental samples (i.e. present in the cDNA libraries).

The CAPP database consisted of more than 2,000 enzyme classes (according to the Enzyme Classification system) where the most common classes were histidine kinase (EC 2.7.13.3) with 14,429 entries, non-specific serine/threonine protein kinase (EC 2.7.11.1) with 10,138, ABC-type vitamin B12 transporter (EC 7.6.2.8) with 8,029, D-inositol-3-phosphate glycosyltransferase (EC 2.4.1.250) with 6,959 and DNA helicase (EC 3.6.4.12) with 6,575 (Appendix C: Table C3). Enzymes in the database included those with industrial relevance, for example, those involved in food processing (e.g. β-galactosidases and α-amylases), in molecular biology protocols (e.g. DNA ligases and alkaline phosphatases) and enzymes used in bioprocessing and bioremediation, such as cellulases and polyphenol oxidases. Contigs belonging to the high- and medium-quality MAGs had 231 complete secondary metabolite biosynthetic gene clusters (BGCs). The most abundant BGCs codified for terpene, Type III Polyketide Synthase (T3PKS), Non-Ribosomal Peptide Synthetase (NRPS) and NRPS-like, bacteriocin, β-lactone and arylpolyene. Eleven phyla were attributed to these BGCs, with the dominant taxa being Acidobacteria, Proteobacteria, Actinobacteria and Chloroflexi (Appendix C: Table C4).

**Figure 4.**
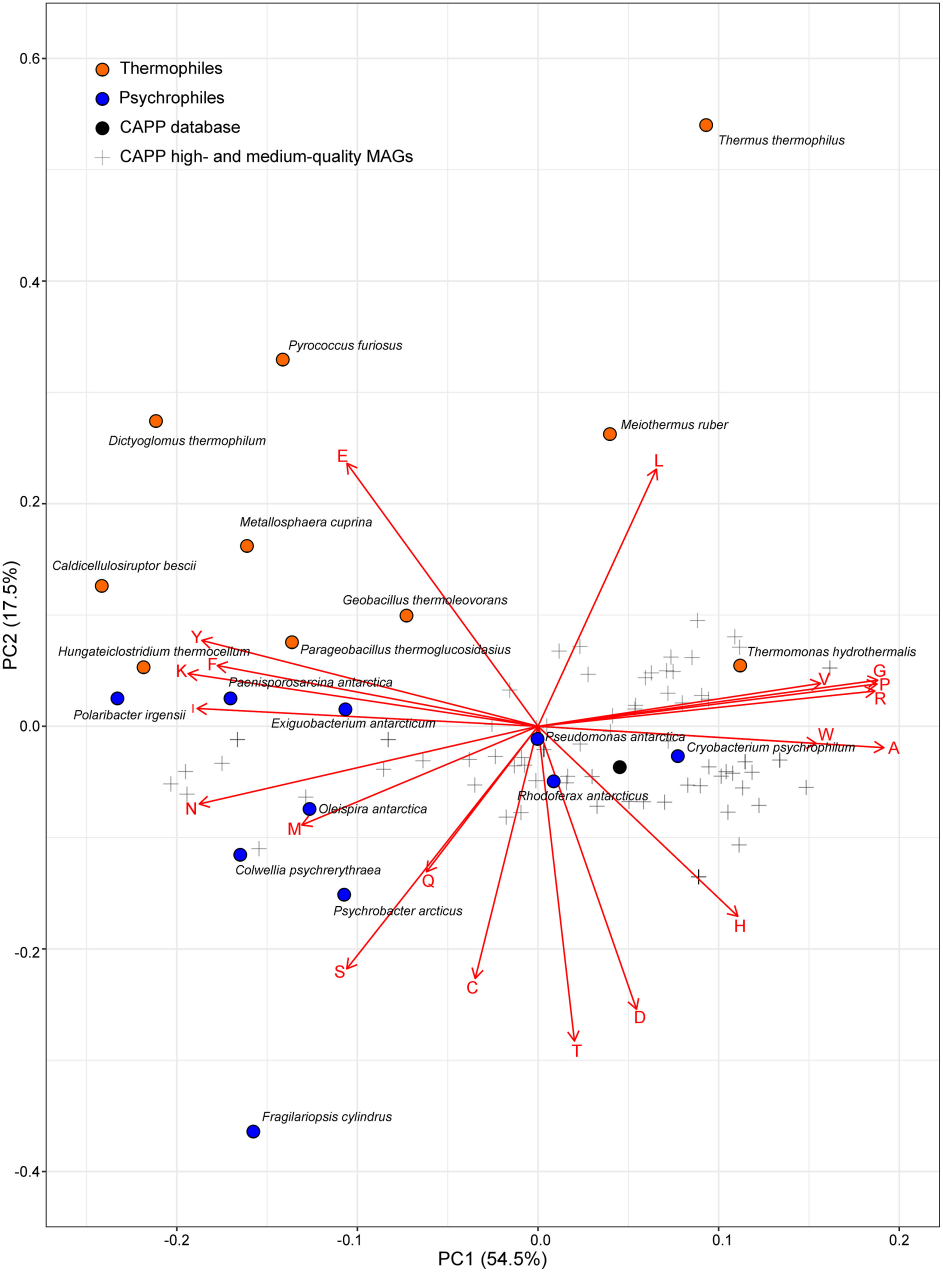
Principal Component Analysis (PCA) of the amino acid composition of proteins from known thermophilic and psychrophilic organisms, from the CAPP database and from the high- and medium-quality MAGs retrieved from the CAPP database. Vectors indicate the direction of the amino acid effect in the proteins' sequence composition specific to each organism and MAG. Eigenvalues are reported in Appendix C (Table C5).

The comparison between the amino acid usage of heat- and cold-adapted proteins obtained from known psychrophilic and thermophilic organisms showed a separation between cold and heat-adapted proteins along the second principal component axis (PC2), explaining 17.5% of the observed variance (Fig. 4). This clustering was driven by an increase of the amino acids glutamic acid (E) and leucine (L) in the heat-adapted proteins and an increase of methionine (M), serine (S), glutamine (Q), cysteine (C), threonine (T), aspartic acid (D) and histidine (H) in the cold-adapted proteins (Fig. 4). The CAPP proteins, together with most of the MAGs’ proteins, clustered closer to the known cold-adapted proteins (e.g. *Rhodoferax antarcticus* and *Cryobacterium psychrophilum*). Some of the MAGs clustered close to *Thermomonas hydrothermalis* (Fig. 4). The majority of the variance was explained along PC1 (54.5%).

**Figure 5.**
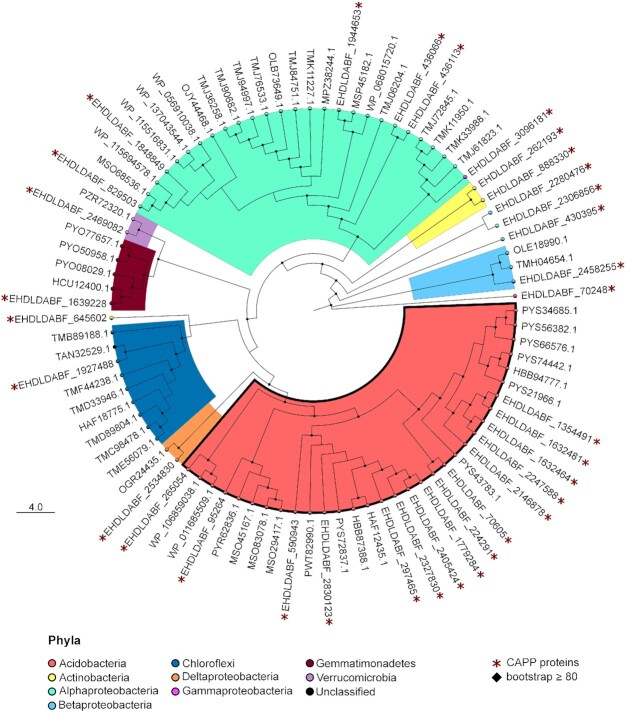
Phylogenetic tree for the protein polyphenol oxidase constructed with both the CAPP database proteins and nr proteins.

To further explore the CAPP database and to identify amino acid substitutions in specific industrially relevant proteins, we further analysed the enzyme polyphenol oxidase (EC 1.10.3.-). The phylogenetic tree built with polyphenol oxidase proteins included 33 and 54 entries from the CAPP and the nr databases, respectively. All the nr entries were from temperate soil and freshwater metagenomes, except from one Antarctic sourced entry from the Verrucomicrobia cluster. The tree sequences belonged mainly to the phyla: Acidobacteria, Proteobacteria (i.e. Alphaproteobacteria) and Chloroflexi (Fig. 5).

**Figure 6.**
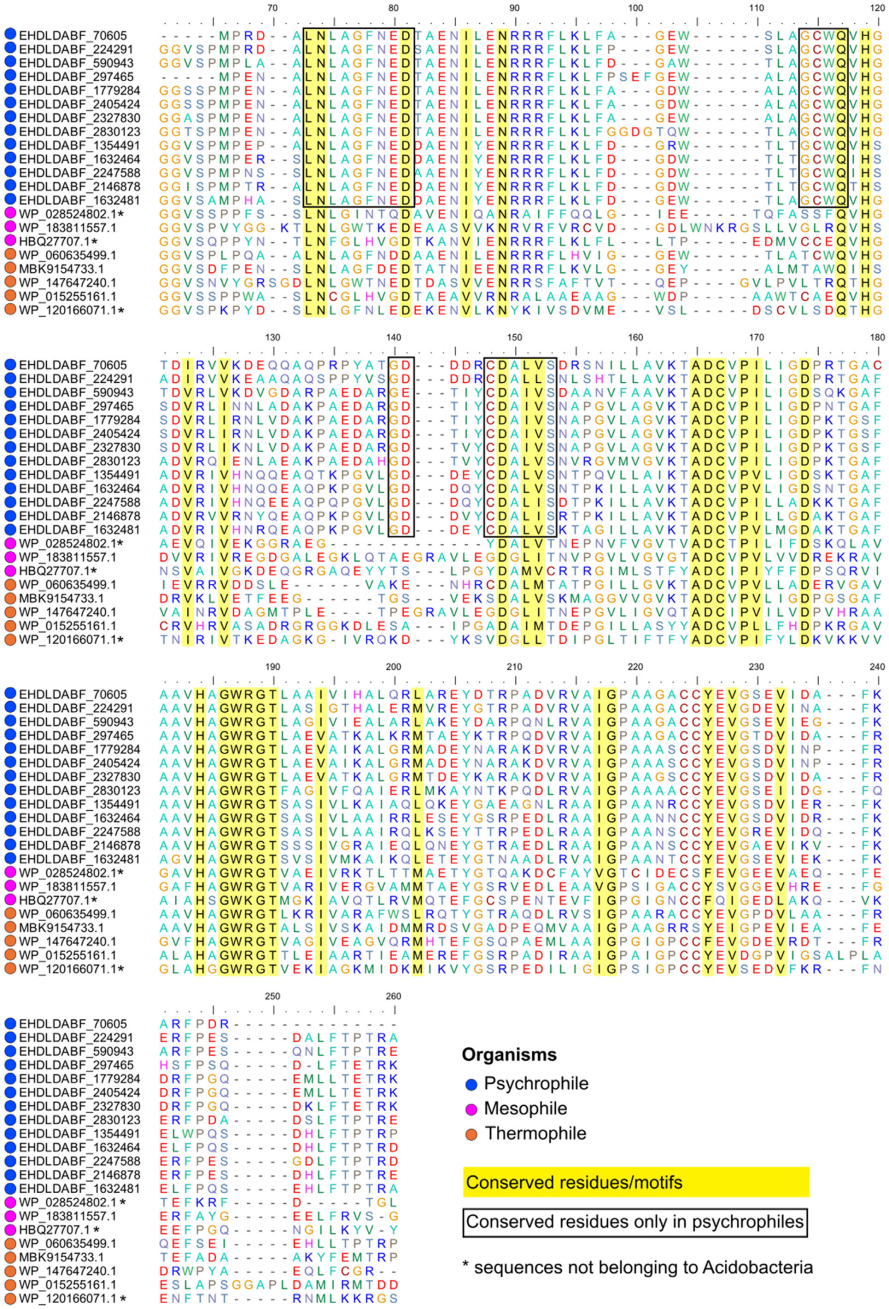
Sequence alignment of the PPO enzyme from thermophilic, mesophilic and psychrophilic bacteria. All the sequences belonged to the Acidobacteria phylum, except from WP_028524802.1 (Bacteroidetes), HBQ27707.1 (Firmicutes) and WP_120166071.1 (Firmicutes). Regions highlighted in yellow are conserved residues/motifs across all the organisms. Regions highlighted in black are residues/motifs conserved only in psychrophilic PPO enzyme. The alignment is reported from position 60 to 260.

We then explored in detail the amino acid frequency in the Acidobacteria cluster (Fig. 5), comprising of 13 complete CAPP protein sequences. The alignment performed with the PPO psychrophilic sequences from the CAPP database, and with mesophilic and thermophilic proteins, showed several protein motifs and amino acid residues conserved across all organisms, and across only cold-adapted PPO protein sequences (Fig. 6). In particular, the sequence alignment of psychrophilic PPO enzymes contained conserved motifs/residues in their N-terminal and C-terminal regions (LNLAGFNED, GCWQ, G(D/E), CDA(L/I) (V/L/I)S). The corresponding region is typically substituted with hydrophobic/uncharged residues in thermophilic PPOs. For example, in the GCWQ motif the cysteine (C) was replaced by hydrophobic alanine (A) or a polar uncharged residue (threonine/serine) while in CDALVS, the (L/I) (V/L/I)S motif was mostly substituted by hydrophobic residues.

## DISCUSSION

We studied permafrost active layer soil samples collected along a transect with varying distance from the Greenland Ice Sheet (GrIS) edge, including a variety of habitats and soils with different geochemical properties. We analysed the microbial community in these samples, using extracted DNA and synthesised cDNA to reconstruct high- and medium-quality MAGs and to create a database of cold-adapted predicted proteins.

### Microbial patterns

GrIS proglacial system permafrost soils showed an active (i.e. cDNA-dependent) and potentially active (i.e. DNA-dependent) microbial community across all samples (Appendix E). Neither the DNA nor cDNA datasets significantly correlated with the sample site distance from the ice edge, and microbial trends, usually observed in surface soil microbiomes in proglacial successions, were not observed (e.g. no decrease in Cyanobacteria and Bacteroidetes with increasing distance from the ice edge was noted) (Schmidt *et al*. 2008; Liu *et al*. 2012; Fernández-Martínez *et al*. 2017). We suggest that the frozen soil environment could create a consistent community of organisms capable of surviving the environmental constraints in the broad variety of sampled sites, from thermokarst bogs to *Salix* heath. These communities may comprise both the non-active organisms trapped in the dark and cold permafrost at its formation, as an impermeable layer isolated from the rest of the soil horizons (Woo *et al*. 2008), and also the more active cold-adapted community shaped by environmental selection factors set by this challenging environment (Ren *et al*. 2018; Malard *et al*. 2019). In fact, both DNA- and cDNA-derived phylogenetic datasets showed a significant correlation with the geochemical variables, where the cDNA trends showed a stronger statistical correlation compared to the DNA dataset (*r* = 0.40 and 0.22 for the cDNA and DNA datasets, respectively). Additionally, more taxa in the cDNA dataset correlated to geochemical variables (Fig. 3), where the putative active taxa were strongly impacted by factors such as nutrients (e.g. DON and DOC) and ion concentrations (e.g. magnesium, calcium and iron) and where, for example, the latter can directly influence and limit the microbial activity serving as enzyme cofactors (Pasternak, Kocot and Horecka 2010; Miethke 2013; Salama *et al*. 2020). While we acknowledge that mRNA extracted from organisms living at subzero temperatures may not fully represent the active microbial community because of the possible preservation of the RNA in frozen conditions (Gadkari *et al*. 2020), we suggest RNA/cDNA profiles are, at the very least, indicative of an active microbial community in frozen soils.

Most of the taxa identified in our permafrost samples have been previously found in this environment (Steven *et al*. 2006; Jansson and Taş 2014). The most active organisms in our dataset, belonging to Actinobacteria, Acidobacteria, Proteobacteria, Chloroflexi, Verrucomicrobia and Gemmatimonadetes (Fig. 2), have previously been identified as active at a wide range of subzero temperatures using stable isotope probing (Tuorto *et al*. 2014; Gadkari *et al*. 2020). Furthermore, tundra soil organisms belonging to Ignavibacteria, *Candidatus* Saccharibacteria, Verrucomicrobia, and Proteobacteria (e.g. Burkholderiaceae) increased in relative abundance and activity when incubated at subzero temperatures (Gadkari *et al*. 2020). Whereas Verrucomicrobia and the Proteobacteria family Burkholderiaceae were present in our dataset (Figs. 2 and 3), Ignavibacteria and *Candidatus* Saccharibacteria were poorly represented (0.10% ± 0.06% and 0.28% ± 0.42% in the DNA dataset and 0.06% ± 0.02%, and 0.17% ± 0.11% in the cDNA dataset, respectively). In the permafrost, organisms are present at different metabolic states (Steven *et al*. 2006; Jansson and Taş 2014). Organisms that were predominant in the DNA but not in the cDNA datasets (e.g. Gammaproteobacteria or Firmicutes) could be in a state of dormancy and exit this state only when more favourable environmental conditions are present (Lebre, De Maayer and Cowan 2017; Jansson and Hofmockel 2018).

### CAPP database

The assembly of the full metagenomic dataset yielded 69 high- and medium-quality MAGs and 213 complete biosynthetic gene clusters (BGCs), highlighting the good assembly quality. The BGCs identified in this dataset comprised gene clusters for the putative biosynthesis of ribosomally synthesized peptides, such as the bacteriocins, nonribosomal peptides (NRP), synthetized by non-ribosomal peptide synthetase (NRPS), and also polyketides (PK), synthesized by Type I and III polyketide synthases (T1PKS and T3PKS). These compounds (i.e. bacteriocins, NRPs and PKs) are widely used in the pharmaceutical industries as antimicrobial compounds and drugs (Shen 2003; Du and Lou 2010; Egan, Ross and Hill 2017). In our database, many organisms were found to possess these clusters (Appendix C: Table C4) indicating that permafrost communities may be excellent resources for BGC biodiscovery.

The microbial genomes of the permafrost community were also used to create the Cold-Adapted Predicted Protein (CAPP) database. Further to report cold-adapted protein sequences, i.e. proteins reconstructed from psychrophiles (Jansson and Taş 2014) (Figure 4), in order to explore the cold adaptability of these proteins, this database reports which of the protein transcripts were potentially transcribed at subzero temperatures, and the observed geochemical conditions (e.g. ion and nutrient concentrations). This database, and similar databases, could be used in the protein engineering field to explore amino acid differences between psychrophilic and mesophilic proteins, and to take an informed approach towards single amino acid protein modifications to create proteins active at lower temperatures. Some amino acids can have different stabilising properties on protein structures where, for example, some have been shown to facilitate protein structure stabilisation (e.g. hydrogen bonds and salt bridges), therefore reducing protein structural flexibility at close-to-freezing point conditions (Åqvist, Isaksen and Brandsdal 2017; Margesin and Collins 2019; Chao *et al*. 2020). However, amino acid sequences and substitutions between homologous proteins (i.e. cold-adapted and heat-adapted proteins) are highly protein and position specific where even one amino acid substitution can lead to protein malfunction and to unbalanced trade-off between protein activity and stability (Siddiqui and Cavicchioli 2006; Siddiqui 2015). This highlights the importance of adopting a protein-focused approach for protein modification.

In comparing amino acid compositions of the CAPP proteins with known psychrophilic and thermophilic proteins, it was evident how the clustering between cold- and heat-adapted organisms was only a secondary factor (Fig. 4), and that the differential amino acid frequencies of these genomes were also shaped by other unidentified factors (e.g. other geochemical variables or taxonomic preferential amino acid usage). For this reason, we focused on a comparison of polyphenol oxidases, comparing enzymes of the CAPP database with those derived from organisms isolated from temperate and hot environments.

Polyphenol oxidizes are enzymes belonging to a group of copper containing metalloproteinase and are members of oxidoreductases that catalyse the oxidation of a wide range of phenolic compounds by utilising molecular oxygen (Kamal-Alahmad, Gasmalla and Alyousef 2015). Alignment of PPO enzyme sequences from thermophilic, mesophilic and psychrophilic bacteria showed considerable homology among each other, with the identification of several conserved motifs (Fig. 6). In particular, all PPOs consist of histidine residues that are important for the stabilization of two copper ions of the active site in the structure, and of cysteine residues, important in the formation of thioether bridge for the protein activity. These residues are involved in maintaining active site conformation by keeping metal cofactor in place (Motoda 1999) and were well conserved across all bacterial PPO enzyme, independently of their thermo-adaptive nature (Fig. 6).

Comparisons of the psychrophilic PPO sequences with mesophilic and thermophilic sequences also highlighted conserved motifs specific to cold-adapted PPO proteins only (Fig. 6). However, no common amino acid substitutions were observed between the different thermo-adapted organisms. Nevertheless, the identification of sequence motifs specific to the cold-adapted PPO sequences demonstrates the wider value of the CAPP database as a resource for identification of sequence dependent regions or locations which may have relevance to low temperature-dependent protein properties such as cold-activity or thermolability.

## CONCLUSION

This is a two-part study where we combined two key aspects of microbial exploration: the study of microbial diversity and structure, and the biodiscovery of new microbial proteins. In the first part we characterised the active layer microbial community along a proglacial transect in southwest Greenland. Here, even though the sampling areas comprised different environments (e.g. thermokarst bogs and grasslands), the microbial community showed a consistent taxonomic composition and did not show microbial trends typical of the proglacial systems but, possibly, a microbial community shaped by the challenging conditions set by the permafrost environment. This indicates the frozen soil environment (e.g. permafrost) as a promising environment to allow in-depth biodiscovery exploration. In the second part, the high-quality deep coverage assembly was used to reconstruct high-quality MAGs and to create the CAPP database. The CAPP protein sequences can be used to explore amino acid substitutions between known thermophilic, mesophilic and psychrophilic proteins with the aim to look at position- and taxon-specific amino acid substitutions; therefore informing protein models that aim to lower the optimal enzymatic reaction temperature of mesophilic proteins, bringing advantages in the industrial and bioremediation sectors. This database is only a first step towards protein studies, and further protein investigations (e.g. gene cloning) are needed. To conclude, we suggest the need to create a public cold-adapted database to easily deposit and explore this kind of data as, we believe, it could lead to more efficient and easy protein modification protocols.

## AUTHOR'S CONTRIBUTION

GV and GB designed the work and collected the soil samples. GV and AS performed DNA and RNA extraction and nanopore sequencing. MR and FS performed the geochemical analyses. SS explored polyphenol oxidase enzyme and performed the sequence alignment. GV wrote the original draft of the article. GV, GB, MR, FS, SS, AS and DC revised and edited the manuscript.

## Supplementary Material

fiab127_Supplemental_Files
