# Supplementary material for: Microbial characterisation and Cold-Adapted Predicted Protein (CAPP) database construction from the active layer of Greenland's permafrost
Source: FEMS Microbiol Ecol. 2021 Sep 1;97(10):fiab127. doi: 10.1093/femsec/fiab127 (PMC8445667; doi:10.1093/femsec/fiab127)
Supplement: fiab127_Supplemental_Files [file fiab127_supplemental_files.zip › Appendix_D_Supplementary_Data.docx]

**Figure D1.** Geochemical trends across the proglacial sampling sites. Values are shown as mean ± standard deviation for each site. These figures report the variables that were significantly correlated (p-value < 0.05) to site distance from the ice edge in the Spearman's rank correlation tests (results shown in Table 1).

**Figure D1 continued.** Geochemical trends across the proglacial sampling sites. Values are shown as mean ± standard deviation for each site. These figures report the variables that were not significantly correlated (p-value ≥ 0.05) to site distance from the ice edge in the Spearman's rank correlation tests (results shown in Table 1).
